# Poultry genetic heritage cryopreservation and reconstruction: advancement and future challenges

**DOI:** 10.1186/s40104-022-00768-2

**Published:** 2022-10-09

**Authors:** Yanyan Sun, Yunlei Li, Yunhe Zong, Gamal M. K. Mehaisen, Jilan Chen

**Affiliations:** 1 Key Laboratory of Animal (Poultry) Genetics Breeding and Reproduction, Ministry of Agriculture and Rural Affairs, Institute of Animal Science, Chinese Academy of Agricultural Sciences, Beijing, 100193 China; 2 Department of Animal Production, Faculty of Agriculture, Cairo University, Giza, 12613 Egypt

**Keywords:** Breed reconstruction, Cryopreservation, Germplasm, Gonad, PGC, Poultry, Semen, Somatic cell

## Abstract

Poultry genetics resources, including commercial selected lines, indigenous breeds, and experimental lines, are now being irreversibly lost at an alarming rate due to multiple reasons, which further threats the future livelihood and academic purpose. Collections of germplasm may reduce the risk of catastrophic loss of genetic diversity by guaranteeing that a pool of genetic variability is available to ensure the reintroduction and replenishment of the genetic stocks. The setting up of biobanks for poultry is challenging because the high sensitiveness of spermatozoa to freezing–thawing process, inability to cryopreserve the egg or embryo, coupled with the females being heterogametic sex. The progress in cryobiology and biotechnologies have made possible the extension of the range of germplasm for poultry species available in cryobanks, including semen, primordial germ cells, somatic cells and gonads. In this review, we introduce the state-of-the-art technologies for avian genetic resource conservation and breed reconstruction, and discuss the potential challenges for future study and further extending of these technologies to ongoing and future conservation efforts.

## Introduction

Over the centuries of domesticated chicken breeds formation, they have undergone genetic evolution underlying unique behavior, morphology, reproduction and adaptations traits [1]. This made them precious local varieties of cultural heritage. Moreover, this genetic diversity permits future integration of indigenous breeds into commercial breeding programs for future exploitation of potentially important traits following the upward trend in the demand, such as diversified flavor and quality, disease-resistance, and climate-specific traits.

The chicken has been the world’s most numerous domestic animals. Globally, more than 60 billion chickens are raised annually to produce around 100 million tons of chicken meat and 70 million tons of eggs for food consumption [2, 3]. Despite their popularity and ubiquity, the biodiversity security is facing challenges in international scope. A very narrow range of standard breeds are used under intensive genetic selection and crossed via the pyramidal structure to produce this vast number of commercial hybrid birds in industry systems. Breeding targeted at genetic uniformity and highly productive individuals further narrows the gene pool and impoverishes allele polymorphism. It is reported that in those commercially pure lines, one-half of the genetic diversity has been contracted because of generations of selection for desirable traits and inbreeding [4]. Furthermore, there is little incentive for local industry producers to develop systematic breeding practices, and indiscriminate crossbreeding also contributed to the erosion of local genotypes. According to FAO statistics, there are 1641 chicken breeds worldwide and 19% of them are endangered, vulnerable, or already extinct. The proportion of avian breeds of unknown risk status is greater than that for mammals (64% vs. 59%) [5]. Many unique specialized experimental poultry lines developed by institutions and universities for fundamental research in biology, biomedicine, and agriculture have been eliminated [6, 7]. Therefore, this genetic diversity crisis in chickens is in wide scope including commercial lines, indigenous breeds, and experimental lines.

The primary efforts to curb the loss of genetic resources have been directed to long-term support for live population maintenance (in vivo) and further research on cryopreservation technology (in vitro). The former still holds a danger of biodiversity loss because of fluctuations and deleterious inbreeding in a small population and the risk of infectious disease outbreaks or environmental disaster [8, 9]. It is imperative that existing germplasm be stored cryogenically in vitro to halt the current drain and rescue extant resources.

Germplasm refers to the germ cells as well as their precursors bearing heredity. With the development and maturity of cryobiology methods and assisted reproductive technology, the establishment of biobanks for cryopreservation of germplasm enables sustainable and economical maintenance of animal genetic resources. The feasibility of developing in vivo cryopreservation collections varies among species. Poultry presents a unique challenge owing to some biological factors. Firstly, poultry semen is highly sensitive to the cryopreservation, coupled with the hen being the heterogametic sex. Secondly, cryopreservation of the ovum and embryo of macrolecithal species is unfeasible due to the large size and high quantity of lipid deposition of the eggs. The recent advances of cryopreservation and breed reconstruction technique in poultry with semen, stem cells, somatic cells, and gonads brings prospect for biobanking, although each having clear differences in limitations in terms of practicality, feasibility, efficiency, and cost. This review examines these emerging technologies that can be applied in the coming decade for increased sustainability of poultry genetic resources.

## Semen

Cryopreservation of semen was firstly succeeded in chickens more than 70 years ago, with the serendipitous discovery of glycerol as the cryoprotectant (CPA) and initiated the cryobiology thereafter [10]. However, the development in poultry has largely lagged behind that for mammals and represents for a long-standing technical challenge. The inherent characteristics unique to poultry spermatozoa likely influence to a large extent the outcome of semen cryopreservation [11]. The poultry spermatozoa are lanceolate or filiform-shaped and have a relatively lower surface area-to-volume ratio and less cytoplasm [12]. Furthermore, the poultry has longer (80–90 μm) and thinner flagellum than the mammalian one, conferring its susceptibility to injury from mechanical manipulations [13].

**Fig. 1:**
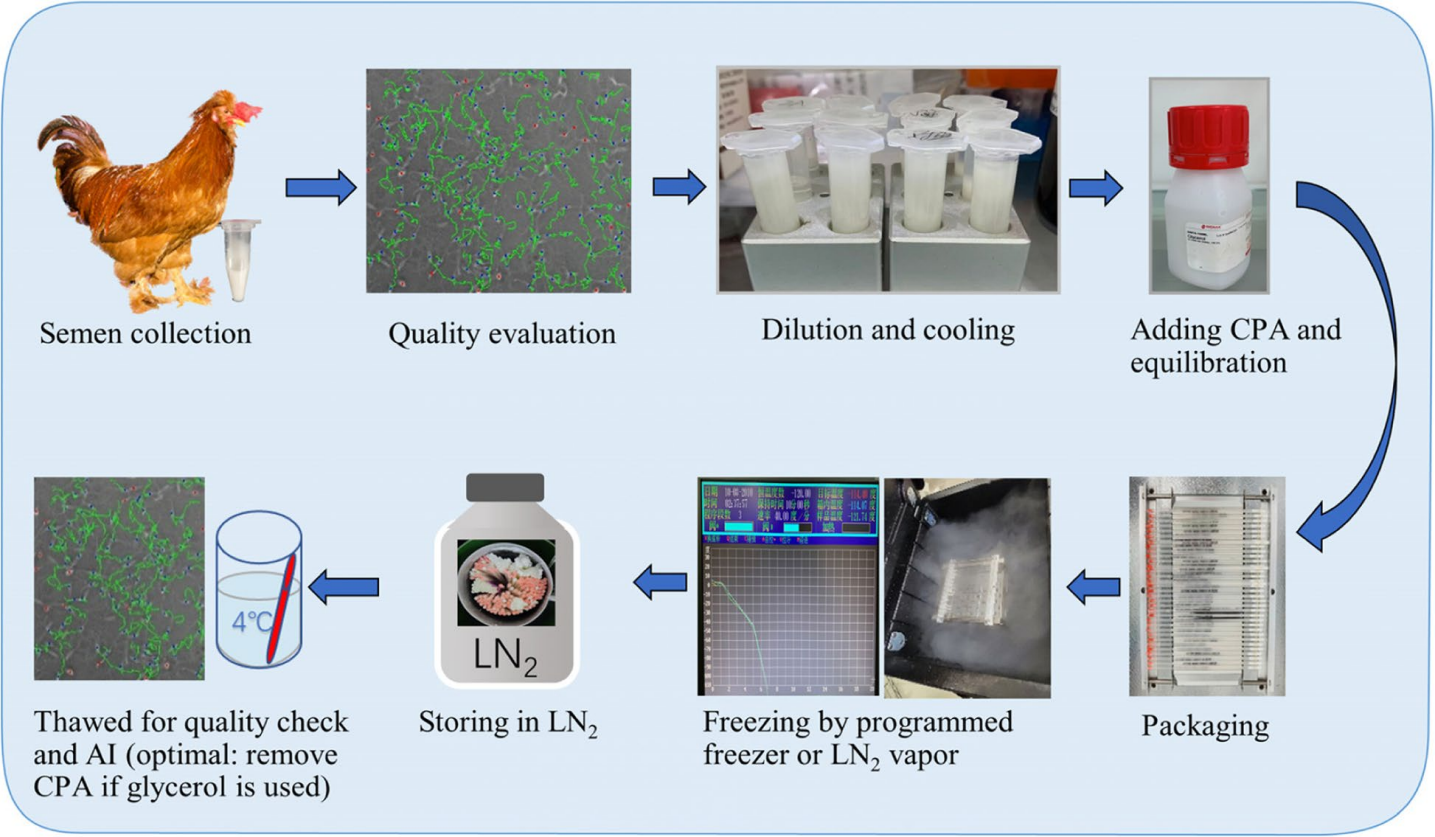
Fundamental steps of freezing protocol for poultry semen

The earlier detailed freezing techniques of poultry semen were developed thanks to the pioneer work of Lake and Stewart [14] and Sexton [15]. The steps for any freezing protocol include semen collection, evaluation, dilution, cooling and equilibration, adding CPA, freezing, storage in liquid nitrogen (LN_2_), and thawing for usage (Fig. 1). Semen extender, CPAs, cooling conditions, equilibration duration, freezing and thawing rates, and packaging type are the key points obtaining the most research interest. In the lengthy process, a constant improvement is continuously made to define the best and standardized protocols to promise higher fertility with the high-valued banked semen.

### Sperm cryoinjury and CPAs

**Fig. 2:**
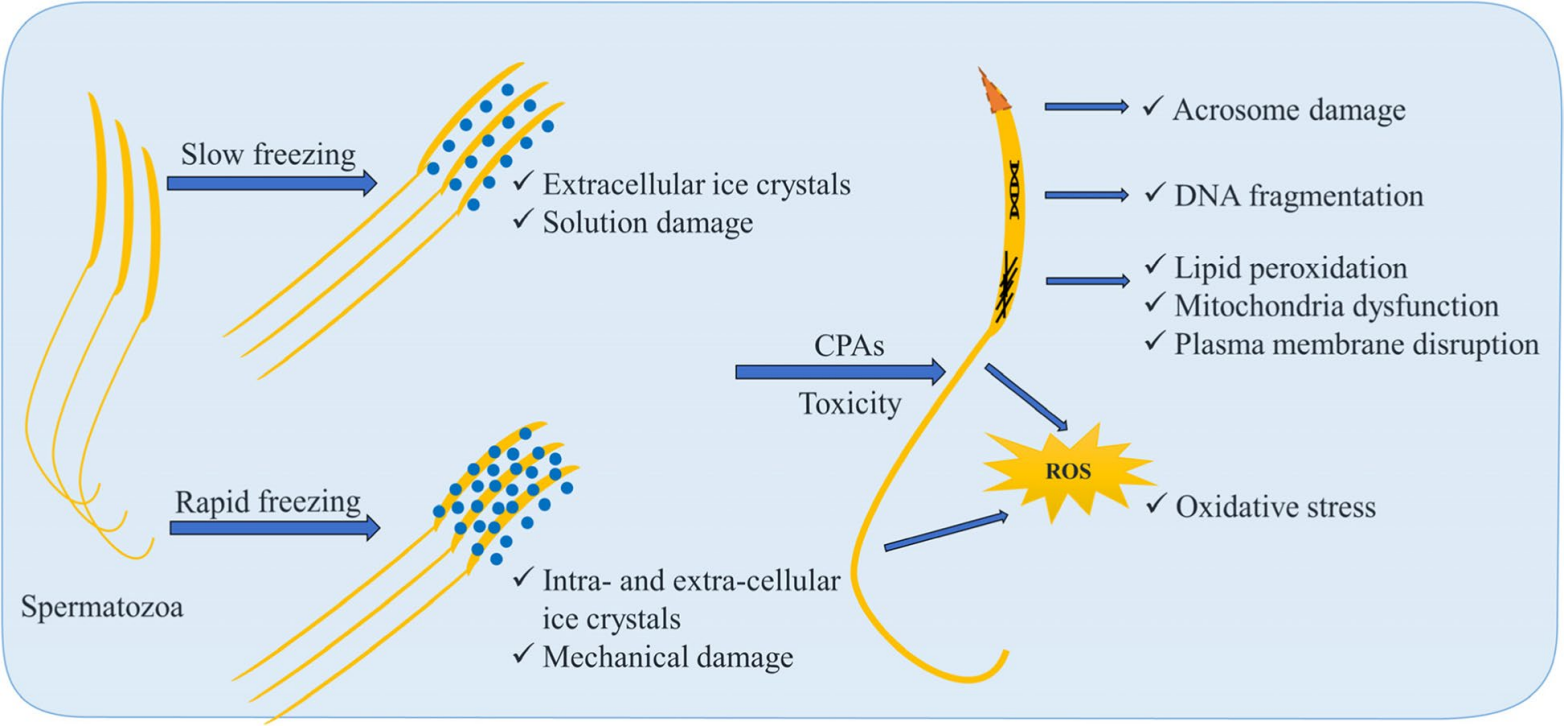
Cryoinjury mechanisms involved during freezing–thawing process of poultry semen

Understanding the cryoinjury mechanisms involved during the freezing–thawing process is crucial for systematic optimization (Fig. 2). The major steps of cryopreservation may lead to damages of cell structure, solution effect, oxidative stress, and reorganization of membranes lipids and proteins [16]. Some CPAs are more toxic than others and their toxicity also depends on the concentration [17].

The spermatozoa traverse the lethal intermediate zone of temperature (− 0 °C to − 60 °C) during the slowing freezing and during the thawing process as well, since ice forms spontaneously in the external medium induces both chemical and mechanical damage. The spermatozoa’s contents remain unfrozen and supercooled. The intracellular supercooled water flows out of the cell osmotically because of the greater chemical potential and freezes externally. The water flux across the cell membrane can induce intracellular ice formation, causing mechanical injury to the plasma and organelle membrane and eventually leading to cell death [18]. Extracellular ice also directly damages the cells by puncturing or crushing cell membranes [19]. The species and cell type specific proper cooling rates are therefore crucial in a way that cooling too quickly leads to lethal intracellular ice formation, whereas cooling too slowly makes the cells suffering lethal “solution damage” [20].

These damages are driven directly or indirectly by ice formation during the transitions between normothermic and low temperature [17]. Intracellular or permeable CPAs may diffuse through cell membrane to minimize cell damages by inhibiting the intracellular ice crystals. The most studied intracellular CPAs for poultry semen are glycerol, dimethylacetamide (DMA), dimethylformamide (DMF), and ethylene glycol (EG) [21, 22]. Semen extender, cooling condition, equilibration duration, freezing and thawing rates, and packaging type are normally developed as CPA specific (Table 1). In this review, the most verified two intracellular CPAs glycerol and DMA were discussed in detail.

**Table 1:** The main parameters in chicken semen cryopreservation developed as internal CPA specific

Internal CPA	Extender	Cooling condition	Equilibration duration	Freezing rates	Thawing	Packaging	AI frequency	AI dose	Egg collection (day post AI)	Mean fertility	Reference
Glycerol	Lake PC	4 °C, 15 min	10 min	–7 °C/min	4 °C, 3 min	Straw	Every 4 d	400 × 10^6^	d 2–5	83.3%	[22]
	Lake	4 °C, 30 min	30 min	4 °C to –35 °C at 7 °C/min, –35 °C to –140 °C at 20 °C/min	4 °C, 3 min	Straw	Every 3 d	300 × 10^6^	d 2–4	76.0%	[23]
	Lake	5 °C, 20 min	30 min	4 °C to –35 °C at 7 °C/min, –35 °C to –140 °C at 20 °C/min	5 °C, till thawed	Straw	Every 3 d	(250–350) × 10^6^	d 2–4	63.9%	[24]
	Lake	5 °C, 20 min	1 min	4 °C to –35 °C at 7 °C/min, –35 °C to –140 °C at 20 °C/min	5 °C, till thawed	Straw	Every 3 d	(250–350) × 10^6^	d 2–4	53.7%	[24]
DMA	FEB	4 °C, 15 min	2 min	–60 °C/min	40 °C, 5 s	Straw	Every 4 d	400 × 10^6^	d 2–5	35.3%	[22]
	Lake	–6 °C, 20 min	1 min	dropped directly in LN_2_	60 °C, till thawed	Straw	Every 3 d	300 × 10^6^	d 2–4	88.0%	[23]
	Lake	–6 °C, 20 min	1 min	dropped directly in LN_2_	60 °C, till thawed	Pellet	Every 3 d	(250–350) × 10^6^	d 2–4	92.7%	[24]
	Lake	5 °C, 20 min	1 min	dropped directly in LN_2_	60 °C, till thawed	Pellet	Every 3 d	(250–350) × 10^6^	d 2–4	84.7%	[24]
	Lake	5 °C, 20 min	1 min	4 °C to –35 °C at 7 °C/min, –35 °C to –140 °C at 20 °C/min	5 °C, till thawed	Straw	Every 3 d	(250–350) × 10^6^	d 2–4	26.7%	[24]
	Lake	NA	10 min	5 cm above LN_2_ vapor for 12 min	37 °C, 30 s	Straw	Every 3 d	300 × 10^6^	d 3–5	33.6%	[25]
	Lake	NA	10 min	4 °C to –35 °C at 7 °C/min, –35 °C to –140 °C at 60 °C/min	37 °C, 30 s	Straw	Every 3 d	300 × 10^6^	d 3–5	40.7%	[25]
	Lake	NA	10 min	5 °C to –180 °C at 60 °C/min	37 °C, 30 s	Straw	Every 3 d	300 × 10^6^	d 3–5	24.5%	[25]
DMF	BHSV	4 °C, 15 min	4 min	–15 °C/min	4 °C, 3 min	Straw	Every 4 d	400 × 10^6^	d 2–5	64.8%	[22]
	Schramm	4 °C, 30 min	30 min	–1.3 °C/min from 5 °C to –35 °C, –20 °C/min from –35 °C to –140 °C	20 °C, 3 min	Plastic vials	Every 3 d	300 × 10^6^	d 2–4	79.0%	[23]
	BHSV	4 °C, 60 min	15 min	11 cm above LN_2_ vapor for 12 min and 3 cm above for 5 min	5 °C, 5 min	Straw	Every 7 d	200 × 10^6^	d 2–8	73.4%	[26]
	Schramm	4 °C, 60 min	15 min	11 cm above LN_2_ vapor for 12 min and 3 cm above for 5 min	5 °C, 5 min	Straw	Every 7 d	200 × 10^6^	d 2–8	86.9%	[27]
EG	Kobidil +	5 °C, 30 min	45 min	5 cm above LN_2_ vapor for 15 min	5 °C, 2 min	Straw	NA	NA	NA	NA	[21]
	BHSV	4 °C, 15 min	10 min	–1 °C/min	4 °C, 3 min	Straw	Every 4 d	400 × 10^6^	d 2–5	1.5%	[22]
	Lake	NA	NA	4.5 cm above LN_2_ vapor for 30 min	5 °C, 1–2 min	Straw	Every 5 d	200 × 10^6^	d 2–6	69.0%	[28]

Glycerol is a typically used CPA for chicken semen cryopreservation [22]. The 11% glycerol (~ 1.5 mol/L) is mostly suggested in the literature [22, 29], while lower concentration of 5%–7% is also suggested [30]. Studies repeatedly showed that the level of glycerol required in the freezing medium for successful protection of sperm against freeze-damage is cytotoxic and highly detrimental to fertility [29]. A modification of sperm acrosome reaction capacity [31], and impaired transit of sperms through the oviduct [32] have been proposed to be associated with the contraception. Although the precise underlying biochemical mechanism has not been fully established, this undeniably potent contraceptive effect exerted by glycerol requires its proper removal from thawed semen before intravaginal artificial insemination (AI), so as to obtain satisfactory fertility [33]. The maximum glycerol concentration tolerated by the rooster sperm without alternation of fertilizing capacity and sperm penetration was proposed to be 0.75% [32]. Glycerol can be removed by simple dialysis or using commercial dialysis cassettes, discontinuous density gradient centrifugation using Accudenz or Percoll column, and stepwise dilution with the glycerol-free medium followed by centrifugation. These methods inevitably vary in materials cost, time cost, sperm recovery rate, and sperm quality. Comparative studies in general demonstrated that the stepwise dilution method exerts merits in the ability to yield more sperm and with more intact plasma membranes than the Accudenz process, and therefore maximize the use of finitely stored semen [29, 34]. Discontinuous Accudenz gradient method was also used sporadically in studies [35]. At present, stepwise dilutions followed by centrifugation has been the most common operation in cryopreservation practice with glycerol as the CPA. Still, it requires attention to details during the multiple dilutions as osmotic swelling or shrinkage could happen due to the osmotic imbalance [36] and damage during centrifugation.

Alternative CPAs which can remain in the spermatozoa without harming their fertilization process would be an advantage and therefore some work has been done on this subject. The non-contraceptive DMA with satisfying fertility was normally obtained when used in conjunction with pellet. Chalah et al. obtained 88% fertility with 6% DMA [23] and Tselutin et al. obtained higher fertility (92.7%) with 6% DMA as compared to that of 63.9% with 11% glycerol [24]. However, pellet is not suitable for large cryobank programs because of sanitary and feasibility consideration. Woelders et al. later indicated that the DMA (0.6 mol/L) was also compatible with straws when freezing at intermediate-high cooling rates of about 200 °C/min, and yielded high fertility (87.6% with ASG extender and 78.1% with Lake extender) [37]. Santiago-Moreno et al. [25] compared the effect of different freezing rates when using 6% DMA with Lake extender and straw package, and showed that the 2-step medium freezing rate (5 °C to − 35 °C at 7 °C/min and then − 35 °C to − 140 °C at 60 °C/min) was associated with higher percentage of motile spermatozoa, acrosome integrity, sperm movement quality, and plasma membrane integrity, and that the fertility of slow (5 °C to − 85 °C at 10 °C/min), median and rapid freezing rate (5 °C to − 180 °C at 60 °C/min) did not show statistically difference, being averagely 33%. However, Behnamifar et al. showed that DMA is not compatible with straw package and a medium freezing rate, as reflected by the contrasting sperm motility after thawing (52.17% for 8% glycerol vs. 14.08% for 4% DMA) [38]. Tang et al. recently showed that when using 6% DMA with Lake extender, higher fertility (77%) was obtained in the gradual in-straw freezing using a controlled LN_2_ vapor than the pellet method (65%) [39]. Therefore, DMA methods could be a probable strategy in chicken semen cryopreservation only when used after many adjustments.

Extracellular CPAs protect the spermatozoa by mimicking the effects of intracellular solutes in the extracellular space to reduce extracellular ice crystals. The notable examples used in poultry are polyvinylpyrrolidone (PVP) [40], sucrose and raffinose [26]. They are either used alone or together with glycerol and DMF to replace or reduce the concentration of toxic intracellular CPAs. Egg yolk is used to be the classical extracellular CPAs, attributed to their low-density lipoproteins, phospholipids, phosphatidic acid, and vitamin E components [41]. Considering its undefined composition and animal origin, other additives owning similar properties, like egg yolk plasma [42] and soybean lecithin nanoparticles [43], have been tried and reported to be capable of preventing cryoinjury by mitigating the efflux of cholesterol or phospholipids and antioxidant effect as well.

### Functional additive as supplementation of semen extender

The glycerol and DMA as intracellular CPAs play an essential role in preventing the harmful effects of a hypothermal situation and make the cryopreservation a routine procedure. The search for new functional additives targeting avoiding or helping recovering from cryoinjury are necessary and never stop.

#### Antioxidant

The handling procedures in semen cryopreservation leads to oxidative stress as evidenced by the increased production of reactive oxygen species (ROS) and malondialdehyde (MDA) [44, 45]. Mitochondria and the sperm cell membrane are the main sites sensitive to oxidative stress during the sperm freeze-thawing process. The endogenous antioxidant defense system naturally presented in sperm and semen plasma includes glutathione peroxidase (GSH-Px), superoxide dismutase (SOD), catalase (CAT), and natural non-enzyme antioxidants like vitamin A, vitamin C, vitamin E, and glutathione. Some of them are impaired and insufficient to scavenger the excessive production of ROS such as hydrogen peroxide (H_2_O_2_), superoxide anions (O^2−^), and hydroxyl radicals (OH^−^) during the sperm freeze-thawing process. The high level of polyunsaturated fatty acids (PUFA) of the membrane predisposes roosters’ sperm to lipid peroxidation (LPO) in the presence of excessive ROS and other oxidant species [46]. The peroxidation leads to elevated apoptosis, morphological and functional defect, DNA damage, and results with motility and fertility loss at the end [47, 48]. The contents of antioxidant defense system members vary among species and individuals, which might be associated with the freeze-resistance variation. For example, chicken shows the lowest level of SOD activity in sperm when compared with mammals such as human, ram, bulls, and rabbits [49].

Therefore, external antioxidants and free radical scavenger compound have been assumed to be promising extender supplementation [50]. The effectiveness of antioxidants including flavonoid quercetin [51], Mito-TEMPO [52], serine [53], SOD [54], coenzyme Q10 [55], melatonin [56, 57], *L*-Carnitine [58], hyaluronic acid [59], Vitamin E and CAT [60], herbal extracts like Achillea millefolium [61], and N-acetyl-*L*-cysteineare [62] proved to be effective in decreasing oxidative stress level, increasing membrane functionality, acrosome integrity, and motility of post-thawed rooster sperm. Part of them were further confirmed by in vivo fertility tests (Table 2).

**Table 2:** The effect of functional additives on cryopreservation of poultry semen

Additive and proper concentration	Base extender and CPA	Beneficial effects	Fertility (Treatment/Control)	Reference
Flavonoid quercetin, 10 mmol/L	Beltsville extender, 3% glycerol	Increase sperm motility, membrane functionality, and mitochondrial activity	64%/55%	[51]
Mito-TEMPO, 5 and 50 mmol/L	Lake extender, unspecified CPA	Decrease LPO; Increase mitochondria activity, acrosome integrity, and viability	65%/48%	[52]
Serine, 4 mmol/L	BHSV extender, 6% DMF	Decrease LPO; Increase membrane integrity, acrosome integrity, and mitochondria activity	90%/84%	[53]
SOD, 50 U/mL	Modified Beltsville extender, 3% glycerol	Increase sperm motility and velocity	NA	[54]
Coenzyme Q10, 1 and 2 mmol/L	Lake extender, 3% glycerol	Increase sperm viability, motility, membrane functionality, acrosome integrity, and mitochondria activity	62%/42%	[55]
Melatonin, 1 and 10^−3^ mmol/L	EK extender, 6% DMA	Increase plasma membrane integrity, mitochondria activity, and motile sperm cell count	NA	[56]
Melatonin, 0.25 mg/mL	Undefined, 13.5% glycerol	Decrease oxidative stress level, increase acrosome integrity, plasma membrane integrity, and sperm motility	NA	[57]
L-Carnitine, 1 and 2 mmol/L	Beltsville extender, 3% glycerol	Decrease LPO; Increase sperm motility, viability, and membrane functionality	NA	[58]
Hyaluronic acid, 1 and 2 mmol/L	Beltsville extender, 3% glycerol	Decrease MDA; Increase sperm motility and acrosome integrity	65%/40%	[59]
Vitamin E, 5 mg/mL	Modified Beltsville extender, 11% glycerol	Increase sperm motility, viability, and membrane integrity	NA	[60]
CAT, 100 IU/mL	Modified Beltsville extender, 11% glycerol	Increase sperm motility, viability, and membrane integrity	NA	[60]
CAT, 100 µg/mL	Modified Beltsville extender, 3% glycerol	Decrease LPO; Increase sperm motility and viability	NA	[54]
Achillea millefolium, 3 mg/L	Undefined extender, 8% glycerol	Increase sperm motility and viability	NA	[61]
N-acetyl-*L*-cysteine, 5 mmol/L and SOD, 200 U/mL	EK extender, 6% DMA	Decrease LPO; Increase sperm motility, viability, and mitochondria membrane potential	NA	[62]

#### Membrane modifying agents or membrane-stabilizing additives

Except for antioxidants, the effectiveness of membrane modifying agents or membrane-stabilizing additives were also evaluated. Cholesterol-loaded cyclodextrin (CLC) increased membrane integrity and yield better fertility [63]. Poloxamer 188, a nonionic surfactant that protects cells against membrane rupture, exerts a cryoprotective effect on rooster sperm and allows decreasing glycerol concentration in the extender [35]. Moreover, there are other such additives tested in non-avian species, such as α-Linolenic acid (ALA) that improves the plasma membrane fluidity and integrity of frozen-thawed bovine sperm [64]. Their effectiveness in avian specifies still need verification.

#### Ice blockers

As previously mentioned, cells damages during the temperature transition may be attributed to ice formation. Factors affecting lethality of intracellular ice crystals include the size, shape, and location of the ice crystals, and their mechanism of growth are therefore the potential target to manage. Ice recrystallization inhibitors like small molecule [65], nanometer materials [66, 67], and antifreeze proteins (AFPs) have been tried in other cell types and species [68]. AFPs refer to a group of special proteins naturally presented in several insects, fish, bacteria, and plant (e.g. wheat) species exposed to freezing temperatures. AFPs are capable of binding to ice to inhibit ice recrystallization, and interacting with cellular membrane, making them intriguing molecules to be used in cryopreservation protocols [68]. Type III AFP was firstly reported to enhance the fertility of post-thawed rooster semen [69]. Although other ice recrystallization inhibitors have not been investigated in poultry semen, the benefit observed elsewhere does inspire interests in poultry.

#### Clues for new additives from omics studies

Deciphering of the cellular and molecular changes that occur during cryopreservation by modern omics technology provide opportunity for developing new additives. Cheng et al. [70] explored 33 proteins differentially expressed between fresh and frozen-thawed sperm. Of which, 19 proteins including fructose-bisphosphate aldolase C, triosephosphate isomerase, aconitate hydratase, tubulin and outer dense-fiber protein were associated with sperm energy metabolism, hydrolase activity, signal transduction, and flagellum structure. Qi et al. [71] identified 2115 differentially expressed genes between fresh and frozen-thawed sperm in roosters, including antifreeze proteins, and their following addition of heat shock protein 90 (HSP90) did increase sperm viability and motility of post-thawed rooster sperm.

Apart from factors of technique, lines or individual males are likely to vary in the capacity to withstand freezing [12, 72, 73]. Similar observations have also been observed in others such as bulls [74], boar [75], and mouse [76]. This was termed as cryoresistance, cryosensitivity, cryotolerance, freezability and freezing capacity in different studies. The heritability of frozen chicken semen fertility related traits was estimated to be between 0.08 to 0.16 [77]. A reasonable hypothesis is that genetic differences exist in the susceptibility of cellular compartments to freeze damage [36]. It is worth of finding the genetic determinate or biomarkers underling the high resistance of freezing, which may help screening for the new additives from a different aspect. For example, Santiago-Moreno et al. indicated the variation of seminal plasma amino acid contents and a positive relationship between concentration of valine and leucine with frozen-thawing sperm viability and DNA integrity [73]. Targeted enrichment of valine was developed as per and was proved to be effective [78]. Ribeiro et al. showed the genetically determined aquaporins expression difference may render the variation in cryotolerance [79]. These results are important for the identification of potential biomarkers predicting freezability and for the development of new additives and protocols [80].

### Semen banking and breed reconstruction in practice

Despite their similar morphology, the cryosurvival of spermatozoa also varies among avian species. Semen cryopreservation has been developed firstly in chickens and then adjusted in other domesticated birds [81, 82]. Although the present-day fertility that is still low for the industry, it is more than adequate for genetic conservation [83]. Semen have been the main type of poultry genetic materials preserved in the majority of many existing national cryobank programs since the beginning of this century [25, 37, 84, 85].

**Fig. 3:**
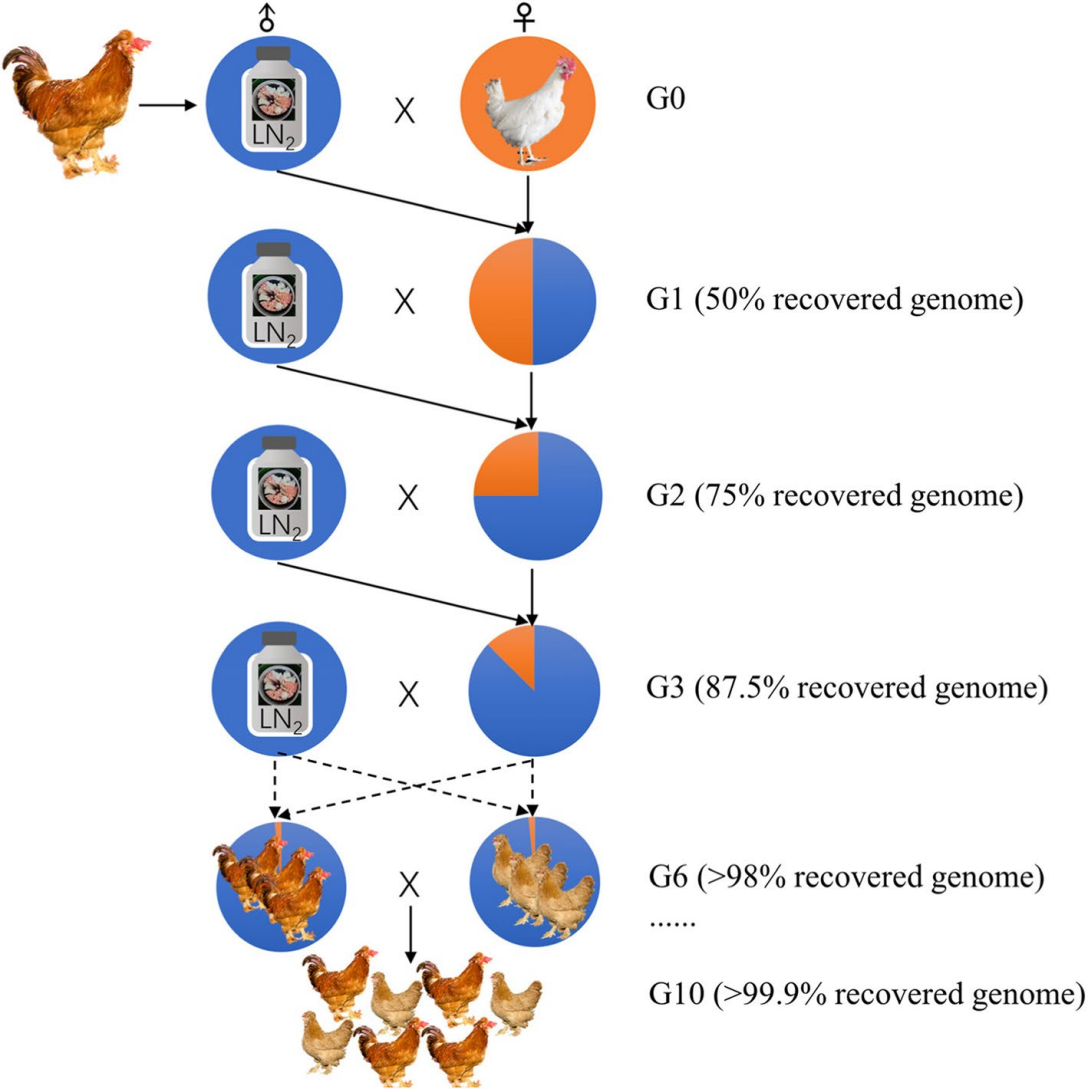
The breed reconstruction using cryopreserved semen

Theatrically, several consecutive generations of back-crossing programs are required to recovery or reconstitution of the nucleus flock of a germ line, as in the case of using only semen. In the classical back-crossing as illustrated in Fig. 3, the cryopreserved semen from the breed that is being recovered is required in each generation to increase the percentage of genes logarithmically. Blesbois et al. encouraged to study other mating design accompanied by molecular methods to restore a line more efficiently [84]. For instance, males and females from the same generation could be intercrossed to produce a population in which genomic molecular markers could be used to select those animals with the highest percentage of the donor genome.

In birds, males are the homogametic sex (ZZ), and females are the heterogametic sex (ZW). The disadvantage of using semen for an avian breed reconstruction is that there is no chance to recover the W chromosome and mitochondrial DNA incorporates variation from the recipient population [86]. Therefore, the continuous efforts are put on the cryopreservation of the complementary genetic materials from the female side.

## Stem cells

**Fig. 4:**
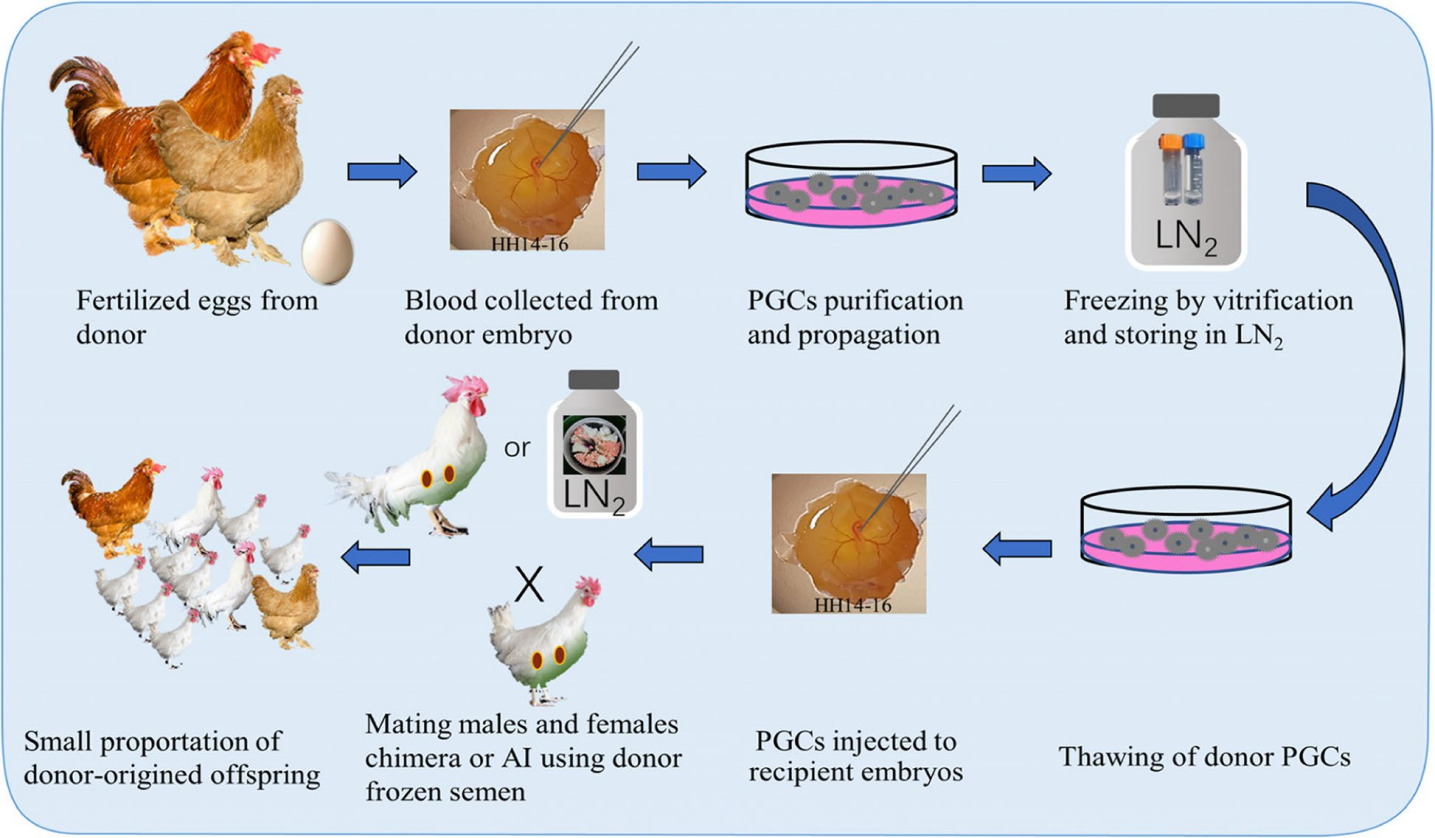
The breed reconstruction using cryopreservation and transplantation of PGCs

Pluripotent germ cells used for alternative cryopreservation of whole genome include diploid blastodermal cells (BCs, which contain limited number of germ cells) from stage X embryos and primordial germ cells (PGCs) from the germinal crescent region or embryonic blood vascular system [87]. Reimplantation of BCs or PGCs into recipient embryos at right stages allows germline chimeric progeny, whose appropriate mating enables the donor breed restoration [88, 89]. This technique was initially achieved in chickens with BCs in 1990 [90] and PGCs in 1993 [91]. The pipeline with PGCs for reconstitution of a threatened chicken breed is shown in Fig. 4. Different from poultry spermatozoa, the BCs and PGCs show good tolerance to both slow freezing and vitrification [92], which may be attributed to their similarity to somatic cells in structure and cryobiology. The expand in culture, survivability of injected recipient embryos, percentage of germline chimeras, and rate of donor-derived gametes from chimeras are the more challenging technique obstacle for stem cells as genetic materials for cryobanks. The accumulated prominent progresses in PGCs in the last decades in cell isolation and culture techniques, and gene-editing technology make this strategy more feasible and practical in poultry genetic resource preservation.

### PGCs isolation

PGCs are the precursors of germline cells scattered in the center of the blastodisc of freshly oviposited eggs. They move passively to the germinal crescent region following the formation of the primitive streak. Thereafter, PGCs circulate through the bloodstream and finally colonize in the gonadal ridge. This unique migration mode allows the accessibility, manipulation, and transportation of PGCs between poultry embryos. PGCs can be readily obtained from the blood (circulating PGCs). The migration of circulating PGCs reaches the peak in the bloodstream between stage 13–17 Hamburger and Hamilton (HH) [93], when is also the optimal time for both collecting donor PGCs and injecting back to the recipient embryo.

Gonad-derived PGCs, also named gonadal germ cells, comprised 2% of the cell from the gonad of day 5–7 embryo [94], and are also capable of re-migrating and differentiating into functional gametes [95, 96]. The gonads are therefore suggested as another ideal collection site for PGCs using the fluorescence-activated cell sorting method [94]. A simple method was later developed by culturing gonads from 7-day-old chick embryos in phosphate buffered saline without Ca^2+^ and Mg^2+^ [97], which showed higher efficiency than the previous proteinases digesting method [96]. Chaipipat et al. [98] recently reported a way of colleting extra PGCs from the culture of embryonic cells following blood aspiration, the so-called tissue PGCs. This maximizes the use of embryonic tissue and meaningful for the resources of limited access [98]. Hu et al. also showed that freezing the entire chicken gonads from embryos of day 8 to 11 post incubation resulted in better germ cells survival and colonization of the host gonad [99].

### Propagation of PGCs in culture

Circulating PGCs comprised less than 0.02% of cells in the blood stream of early embryo, therefore a very small number of PGCs (50–150 cells per embryo) can be expected to be harvested in this way [100]. The peak concentration of circulating PGCs also varies between breeds and affected by incubation temperature [101]. The propagation of PGCs in culture in vitro to expand that small population of cells to 50,000 to 100,000 in 4–5 weeks before cryopreservation is necessary and helpful [102], as it reduces the technical skill needed to isolate and purify the cells before transplantation. This period of culturing also eliminates other cell types and homogeneous cell population can be established. The mutation rate of PGCs was estimated as 2.7 × 10^−10^ de novo single-nucleotide variants (SNVs) per cell division, indicating the remarkably genome stability in the in vitro cultured PGCs [103]. However, the loss of germline transmission competence for the gonadal niche is observed for the PGCs with prolonged culture in vitro (> 3 months) [103]. PGCs survive well after cryopreservation and proliferated robustly when re-cultured after thawing.

### Production of sterile recipients for PGCs transplantation

After the injection into the dorsal aorta or hear of day 2.5 (stage 17 HH) host embryos, the donor PGCs (~ 3000 per embryo) may migrate to the forming gonads and will differentiate into functional gametes in the adult host raised to sexual maturity. Some of the offspring were derived from the exogenous donor PGCs. A major constraint of this system is that because of the presence of endogenous host germ cells competition, the transmission rate from exogenous PGCs injected into the embryo is not stable and generally lower with normal recipients [102]. Infertile recipients, naturally occurring, chemically ablated or genetically engineered, are ideal host for germ cells transplantation [104]. ZZZ triploid chickens are potential recipients but very rare [105]. Surgical removal of BCs or blood from recipient embryos [106], chemical (e.g. busulfan) [107] and physical (e.g. X-irradiation) [108] methods have been adopted to partially ablate the endogenous germ cells of the surrogate host, but the degree is variable and the impeded embryo development induced by the toxicity is non-negligible. Infertile interspecific hybrid between Guinea fowls and chickens was created and evaluated as possible but lower efficiency recipient hosts [109]. Until recently, using genome editing technology, the researchers in Roslin Institute elegantly created genetically engineered sterile female layer chickens as surrogate hosts. The genetic ablation of *DDX4* gene required for germ cell development has been performed to eliminate the endogenous germ cells. This resulted with the observation that all hatched offspring from the chimera genetically engineered hens were derived from the donor, providing a viable platform to conserve and regenerate avian species using cryopreserved PGCs and semen of interested breeds [103]. Their later generation of male sterile chickens permit the resurrection of a poultry breed in a single cross of surrogate host animals [110]. Hu et al. [99] re-established a chicken breed by transplanting the embryonic gonad germ cells to the sterile surrogate host, where 1 out of 7 males hosts generated offspring derived from endogenous host germ cells, and all 12 female hosts produced only donor-originated offspring. It is obvious that the sterile surrogate host increases the proportion of donor-originated offspring and facilitate the breed reconstruction using PGCs. However, it is not in competition with semen use since the two methods are complementary and it is recommended to develop both for the future of genetic resources management.

### PGCs banking in practice

As summarized above, PGCs isolation, culturing, and cell line establishment in vitro, characterization, cryopreservation, reintegration into a recipient embryo can be carried out in well-defined laboratory circumstances. Genetic conservation with PGCs has more and more come into practice instead of only existing at a theoretical level. PGCs cryopreservation of indigenous chicken and quail breeds/lines has begun as part of the National Institute of Agrobiological Sciences Genebank projects in Japan [111]. Successful cryopreservation and regeneration of native chicken breeds using PGCs have been reported recently [112–114]. A uniformly recommended universal protocol based on the latest research finding is therefore in urgent need to facilitate the practice. For example, it was previously widely accepted that the female PGCs in male chicken gonads rarely produce functional spermatozoa [115]. Male and female PGCs should be collected separately for future sex-matched transplantation. While recently, the avian PGCs are proved to be not sexually restricted for functional gamete function. Ballantyne et al. observed that the transplantation of donor circulating PGCs to the opposite sex of sterile surrogate recipients can form functional gamete, i.e. the chromosomally male PGCs (ZZ) formed functional oocytes in the female host, while the female PGCs (ZW) formed functional sperm in the male host [113]. The gonadal germ cells carried by opposite sex host were also proved to be capable of producing functional gametes like the circulating PGCs [99]. This will for sure change the PGCs sampling strategy for the cryobank purpose.

## Somatic cells

### Production of viable chicken from somatic cells

The sexual propagation with somatic cells cloning has been carried out in several agricultural animals such as sheep, goats, pigs, rabbits, and horse. Somatic cells also hold a hope to be reprogrammed into induced pluripotent stem cells (iPSCs) and later induced into reproductive cells capable of fertilization and development to reproductively competent [116, 117]. The prospect of genetic rescue via somatic cell nuclear transfer and in vitro gametogenesis from directed development of iPSCs is receiving growing consideration and obtained significant progress in mouse, species and human [118–120].

It is a milestone achievement in poultry species that a team from Yangzhou University successfully developed a way of producing induced PGCs (iPGCs) from chicken somatic cells and obtained live offspring of donor-origin [121]. They reprogramed chicken embryo fibroblasts (CEF) to iPSCs via introducing expression of four genes-*Oct4/Sox2/Nanog/Lin28A*-system into cells with the addition of vitamin C and valproic acid, and induced iPSCs to develop into iPGCs using BMP4/BMP8b/EGF. After transplantation, those donor’s iPGCs could migrate and home to the genital ridges of allogeneic embryo to produce viable offspring.

### Prospective of cryobanking somatic cells for poultry

Although the complexity and costs when using somatic cells to producing offspring are much greater than other types of germplasm materials, such success still sparks interest in the use of somatic cells (e.g. CEF) or embryo tissues for poultry biobanking. Somatic cells are easily available in large quantities (10^7^ CEFs per chicken embryo) and can proliferate rapidly in vitro. The collection and cryopreservation of somatic cells are therefore relatively simple and cheap. The procedure has to date been accomplished only in chickens in the laboratory and not been developed to applicable across all poultry species. Nonetheless, on a long-time horizon, it offers a convenient back-up solution for collection of poultry genetic resource to be used in the future for reconstituting at-risk or extinct breeds, especially under some conditions with limited access to other germplasm materials and infrastructure present-day. It is hoped that the production of live birds from somatic cells in poultry can develop to a point at which it becomes economical feasible with high success rate, just as the trend we see in pigs for which somatic cells cloning is offered as a commercial service [122].

## Gonad

**Fig. 5:**
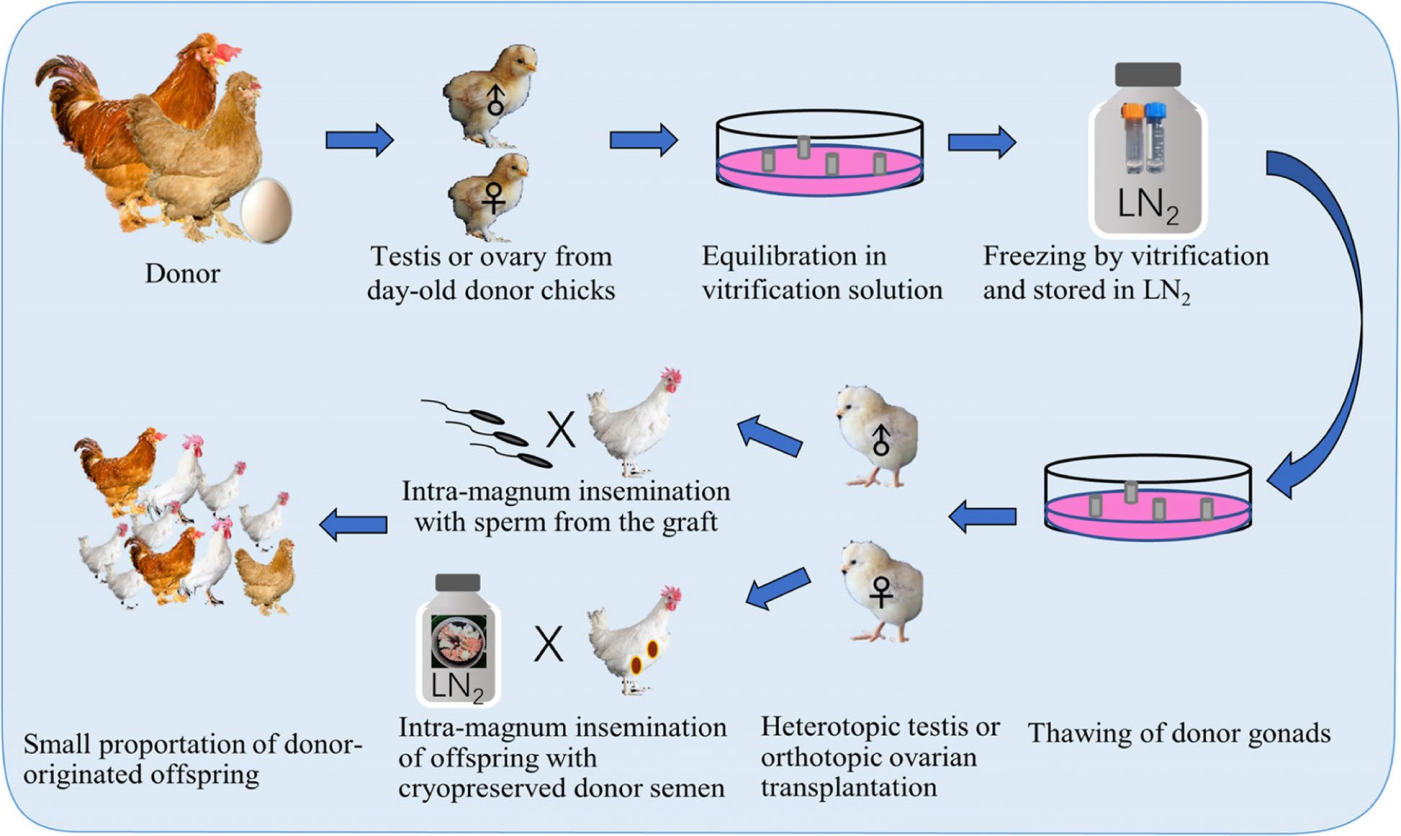
The breed reconstruction using cryopreservation and transplantation of postnatal gonad tissue

The testis and ovary contain spermatogonial stem cells and oogonia and primordial follicles, respectively which are peripherally located and developmentally dormant. Cryopreservation of gonads and functional fertility recovery by transplantation (allograft or xenograft) may therefore provide another option for poultry conservation and regeneration (Fig. 5). The gonads cryopreservation with both slow freezing and vitrification procedures works well as summarized previously [123]. The vitrification method is more recommended in view of its efficiency and simplicity. Significant requirement of surgery manipulation seems to be the most crucial obstacle for this technique. The recent advancement of the allotransplantation technique in poultry added the feasibility of this option, and especially meaningful for the conservation of female poultry genetic pool.

### Cryopreservation and transplantation of postnatal testis

Song and Silversides [124] firstly devised the surgical heterotopic transplantation of testicular tissue fragments, under the skin of the back, under the skin of the abdomen, and on the cranial mesenteric membrane in the abdominal cavity of the surgically castrated and immunocompromised newly hatched recipient chicks. Donor-derived offspring was obtained after surgical intra-magnum insemination of the collected sperm [124]. Based on this recuperation method, they further developed a simple freezing protocol using dimethylsulfoxide (DMSO) as the CPA and a freezing container (− 1 °C/min rate of cooling at − 80 °C) to cryopreserve testicular tissue and obtained offspring with frozen-thawed testicular tissue for the first time [125]. The age of the donors and the cryopreservation methods were optimized. To reduce the mortality of recipients, the castration at 2 or 6 d post hatch and transplantation on the subcutaneous location on the back was suggested [126]. The strategy was further improved by adopting vitrification procedure and extended to other poultry species like Japanese quails [127].

However, the benefit of using testis cryopreservation and transplantation should be critically discussed. In this strategy, the day-old donors are sacrificed to collect the testis, the recipients are executed to collect merely limited amount of sperm from the grafts at the transplantation sites. The intra-magnum insemination is adopted since the collected sperm is almost infertile via normal intravaginal insemination. This surgical insemination further interrupts the egg production of the hens. It is imaginable that the period for breed reconstruction is long and offspring obtained per day-old chick donor is limited. The male donor chicks could have been grown to adults for semen cryopreservation to give more offspring. In this context, this strategy of testis cryopreservation is not that cost-effective. Benesova et al. firstly succeed in transplantation of cryopreserved testicular cells digested from the adult chickens to an irradiated infertile cockerel and obtained donor-derived progeny [128]. Advantages of this strategy lie in the fact that it shortens the period required to obtain sperm, and that the sperm is accessed by dorsal abdominal massage and fertile via shallow intravaginal insemination. However, the efficiency of the testis or testicular cells cryopreservation for male genetic pool conservation is still not competitive with semen cryopreservation.

### Cryopreservation and transplantation of postnatal ovary

Technique of orthotopic ovarian transplantation as an alternative method of artificial reproduction of poultry has been inadequately effective until the success of Song and Silversides in day-old chicks [129] and Kosenko in 30 to 45 days old chicks [130], respectively. The immediate application was carried out in a way that the gonads (both testes and ovaries) of 18 genetically distinct chicken lines from Canadian publicly-funded institutions were cryopreserved using a slow freezing procedure with DMSO as the CPA [131]. Vitrification procedures were employed to cryopreserve gonads from more lines of chickens (day-old), Japanese quail (5 to 7 days old), Chilean tinamou (5-day-old), and turkey (3-day-old) to minimize cellular damage [132–134]. Donor-derived offspring from orthotopic transplantation of cryopreserved ovarian tissue were obtained one after another in Japanese quails [135], ducks [136], and chickens [137]. Worth mentioning is that the Muscovy ducks were obtained from interspecific ovarian transplantation using Pekin ducks as recipients [136].

Theoretically, productive layer breeds selected for egg production are ideal good recipient candidates. Liptoi et al. [137] observed variation of ratio of functioning grafted donor from different donor and recipient breed combinations and proposed that not all genotypes can be used as effective recipients for ovary allotransplantation. Gonads are immune-privileged organs for which the allografting can be successful without delivering of immunosuppressant for the recipient [138]. In the experiment of Liptoi et al., the donor-recipient combination between the same breed showed higher ratio (71% without immunosuppression treatment and 84% with treatment) than those from different breeds (0, with treatment) [139]. The incompatibility was proposed as an excuse. However, this is a paradox to earlier studies which showed successful combinations between breeds when the transplantation is performed with tissue from day-old chicks or from 30 to 45 days old chicks [125, 129, 130, 138]. The governing principle for pairing proper donors and recipients needs further investigation.

On the contrary to males, the procedure of producing donor-originated offspring with female donors is simpler and more meaningful. The donor eggs are feasible over an extended period as soon as the cryopreservation and transplantation were successful, and as high as 50% to 98% of the eggs produced are donor-derived [140]. This might be therefore an especially efficacious alternative in preserving the female poultry genome.

### The application of sterile surrogate host for gonads transplantation

Similar to the work with PGCs transplantation, to increase the ratio of donor-derived to host-derived offspring, the busulfan was used to hinder the growth of host germ cells during the incubation, while the effectiveness or access of busulfan to the embryo is sometimes unpredictable [139, 140]. The ovariectomy of the recipients was performed using forceps or electrocautery which requires great practice for extreme precise and avoid excessive bleeding [137]. None of these two strategies produced offspring solely from donor because of the incomplete removal of the functional recipient ovarian tissue. With the development of genetically engineered sterile recipients, high efficiency is promising.

## Main concerns in the challenges of poultry genetic resources biobanking

The recent upsurge in cryopreservation and reconstitution technology advances of semen, PGCs, somatic cells, and reproductive tissues are meaningful for poultry genetic resources biobanking. There are lingering concerns about the length of time that cells or tissues can be stored in N_2_ vapor phase or in LN_2_ at temperatures below − 150 °C without damage on the viability or development potential. The answer is assumed “indefinite periods”, if no thermally driven normal molecular motion and metabolic reactions occur. Although this has not been exposed for systematical evaluation, some researchers performed preliminary investigation in 13-year sheep embryo [141] and 40-year bull semen [142]. In chickens, Blackburn et al. compared the semen samples frozen in 1986 and 2005 and showed that there was no deterioration of semen once it has been successfully cryopreserved [85]. Thelie et al. also showed that rooster semen straws stored for 18 years in LN_2_ did not lose the fertilizing ability [22]. Therefore, the reasonable time-horizons required for biobanking, if not indefinite, has no detectable effect on the viability of cryopreserved germplasm.

From the aspect of genetic resources conservation, keeping genetic stability and genetic integrity of offspring from cryopreserved collections are crucial challenges. The freezing–thawing process may induce cellular and molecular modifications underlying detrimental effects on genetic stability and integrity [143]. Sperm cryopreservation could entail DNA strand breaks. There is evidence from literature that post-thawed spermatozoa with fragmented DNA may exhibited increased telomere length and altered gene expression in fish embryos [144]. There are also studies reported no evidence of affected human embryo development using cryopreserved semen [145]. In chickens, significant reductions of DNA methylation, H3K9 acetylation and H3K4 methylation as compared to the fresh semen was reported by Salehi et al. [146]. Although cryopreserved semen has been effectively commercially used in livestock for decades, these observation in other species do inspire long-term follow-up studies in chickens on the resultant offspring obtained from cryopreserved materials and thus is highly recommended for future studies to fully assess their biological safety [143]. Combining the above two concerns, it is concluded that the alternations of intrinsic quality of germplasm may most likely happens during the process instead of during a safe storage. The research employing whole-genome sequencing is encouraged to check different types of DNA changes, such as nucleotide substitutions, deletions, and insertions. This may result with more critical appraisal of the evidence regarding the long-term fate of cryopreserved materials and effectiveness of cryopreservation [147].

The second challenge that should be proposed is the collection and smart usage strategy of germplasm. In vivo in situ and in vitro ex situ conservation are complementary rather than mutually exclusive. Silversides et al. indicated that it would be approximately 90% cheaper to cryopreserve the population than maintaining in vivo in situ over a 20-year horizon [86]. Animal genetics resources biobanks were initially conceived to conserve a secure back up germplasm for the worst-case scenarios of reestablishment of breeds facing extinction [148]. The core collection for this purpose is long term and should contain enough germplasm for 150% of regeneration needs, considering the variable viability, fertility, and survival [149]. Beside this typically long-term core collection, a second potential purpose of biobanks could be to support in vivo conservation. Periodic flows of germplasm from genetically different individuals to and from the biobanks for use in in vivo populations is highly recommended [88]. This cryo-aided live scheme may increase diversity and minimize inbreeding by prolonging the generation intervals and genetic drift in small conservation population [150, 151] or selected lines [152]. Short term storage with regular updates of germplasm in the cryobanks under traceable conditions, especially semen, should be arranged to enable this usage. This action can help small populations to get rid of the extinction vortex. To some extent, it also prevents the usage of long-term storage as long as the extinct does not happen. Future challenges will lie in precise integration of these emerging technologies and cryobank collections into existing in vivo systems. Precise means timely action and materials with correct genetic background. These studies have so far been conducted in a very limited scope in poultry. Genetics and genomics tools should be developed for precisely prediction of pros and cons of prospective changes and identifying when, what, and how the cryobank collections are to be explored.

## Concluding remarks

**Fig. 6:**
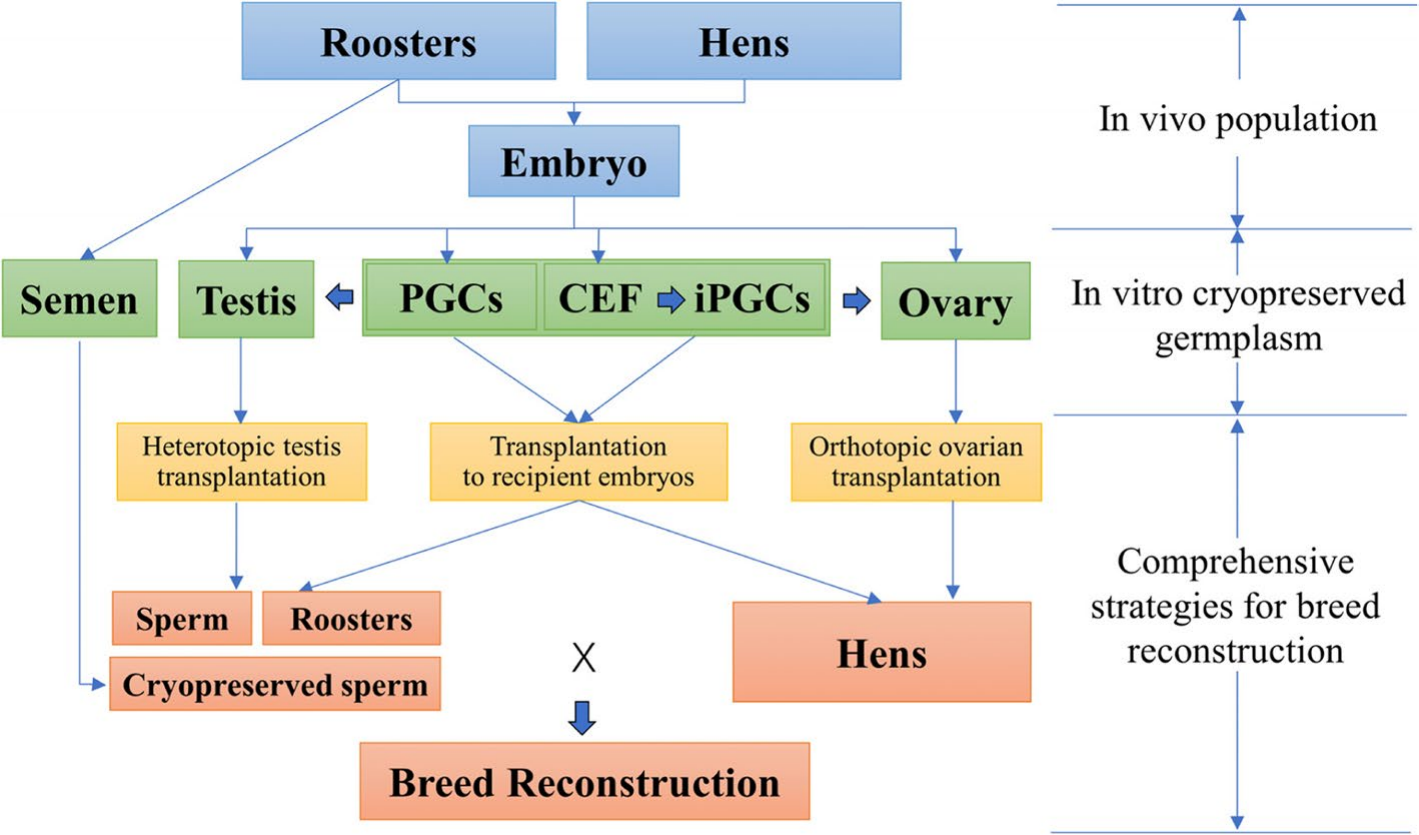
The comprehensive breed reconstruction strategies using multiple cryopreserved germplasm

Animal genetic resources conservation is an interdisciplinary subject, and its effectiveness relays on the endeavor between cryobiology, cellular, reproductive and genetic technologies, and comprehensive information systems [148, 153]. Successful cryobanking of poultry species using the germplasm will have important applications for both ex situ preservation of valuable/endangered breeds and aid efforts to conserve genetic diversity in commercial pure lines, indigenous breeds, and specialized experimental lines. Poultry community has been slow to adopt cryoconservation of germplasm as compared to mammals [126]. According to a recent survey among 15 European and 2 non-European gene banks, 7 reported that they cryopreserved materials for chickens, and that the proportion of breeds with cryopreserved germplasm was below 10% for chickens, and only less than 3% have materials sufficient to allow breed reconstruction [154]. This may also mirror the similar situation elsewhere in the world. Furthermore, it highlights the need for continues efforts in germplasm collection for at risk genetic resources. The prospect quick expanding of poultry germplasm in biobanks may not be at hand very soon, as the new methods need to be standardized before extension. However, the technique advancement documented in the present review do provide an unprecedented opportunity for biobanks to expand the scopes of germplasm for poultry, and partially offer new genetic rescue strategy by concurrent storage and integrated usage of different germplasm and live animals (Fig. 6).

## Data Availability

Not applicable.

## Abbreviations

CPA: Cryoprotectant
LN_2_: Liquid nitrogen
DMA: Dimethylacetamide
DMF: Dimethylformamide
EG: Ethylene glycol
AI: Artificial insemination
PVP: Polyvinylpyrrolidone
ROS: Reactive oxygen species
MDA: Malondialdehyde
GSH-Px: Glutathione peroxidase
SOD: Superoxide dismutase
CAT: Catalase
H_2_O_2_: Hydrogen peroxide
O^2^^−^: Superoxide anions
OH^−^: Hydroxyl radicals
PUFA: Polyunsaturated fatty acids
LPO: Lipid peroxidation
CLC: Cholesterol-loaded cyclodextrin
ALA: α-Linolenic acid
AFP: Antifreeze protein
HSP90: Heat shock protein 90
BCs: Blastodermal cells
PGCs: Primordial germ cells
HH: Hamburger and Hamilto
SNVs: Single-nucleotide variants
iPSCs: Induced pluripotent stem cells
iPGCs: Induced PGCs
CEFs: Chicken embryo fibroblasts
DMSO: Dimethylsulfoxide

## References

[1] Wang MS, Thakur M, Peng MS, Jiang Y, Frantz L, Li M (2020). 863 genomes reveal the origin and domestication of chicken. Cell Res.

[2] FAO, http://www.fao.org

[3] Ritchie H, Roser M. Number of animals slaughtered for meat, World, 1961 to 2018. 2021. https://ourworldindata.org/meat-production#number-of-animals-slaughtered.

[4] Muir WM, Wong GK, Zhang Y, Wang J, Groenen MA, Crooijmans RP (2008). Genome-wide assessment of worldwide chicken SNP genetic diversity indicates significant absence of rare alleles in commercial breeds. Proc Natl Acad Sci U S A.

[5] FAO, Status and trends of animal genetic resources-2018. http://www.fao.org/3/CA0121EN/ca0121en.pdf.

[6] Fulton JE (2006). Avian genetic stock preservation: An industry perspective. Poult Sci.

[7] Fulton JE, Delany ME (2003). Poultry genetic resources-operation rescue needed. Science.

[8] Wilkinson S, Wiener P, Teverson D, Haley CS, Hocking PM (2012). Characterization of the genetic diversity, structure and admixture of British chicken breeds. Anim Genet.

[9] Cendron F, Perini F, Mastrangelo S, Tolone M, Criscione A, Bordonaro S (2020). Genome-wide SNP analysis reveals the population structure and the conservation status of 23 Italian chicken breeds. Animals (Basel).

[10] Polge C, Smith AU, Parkes AS (1949). Revival of spermatozoa after vitrification and dehydration at low temperatures. Nature.

[11] Long JA (2006). Avian semen cryopreservation: What are the biological challenges?. Poult Sci.

[12] Blesbois E (2012). Biological features of the avian male gamete and their application to biotechnology of conservation. J Poult Sci.

[13] Agca Y, Critser JK (2002). Cryopreservation of spermatozoa in assisted reproduction. Semin Reprod Med.

[14] Lake PE, Stewart JM (1978). Preservation of fowl semen in liquid nitrogen-an improved method. Br Poult Sci.

[15] Sexton TJ (1980). Optimal rates for cooling chicken semen from +5 to -196℃. Poult Sci.

[16] Bailey JL, Bilodeau JF, Cormier N (2000). Semen cryopreservation in domestic animals: A damaging and capacitating phenomenon. J Androl.

[17] Bojic S, Murray A, Bentley BL, Spindler R, Magalhes J (2021). Winter is coming: The future of cryopreservation. BMC Biol.

[18] Gao D, Critser JK (2000). Mechanisms of cryoinjury in living cells. ILAR J.

[19] Liu J, Cheng KM, Silversides FG (2013). Fundamental principles of cryobiology and application to ex situ conservation of avian species. Avian Biol Res.

[20] Wilmut I (2014). From germ cell preservation to regenerative medicine: An exciting research career in biotechnology. Annu Rev Anim Biosci.

[21] Miranda M, Kulikova B, Vasicek J, Olexikova L, Iaffaldano N, Chrenek P (2018). Effect of cryoprotectants and thawing temperatures on chicken sperm quality. Reprod Domest Anim.

[22] Thelie A, Bailliard A, Seigneurin F, Zerjal T, Tixier-Boichard M, Blesbois E (2019). Chicken semen cryopreservation and use for the restoration of rare genetic resources. Poult Sci.

[23] Chalah T, Seigneurin F, Blesbois E, Brillard JP (1999). In vitro comparison of fowl sperm viability in ejaculates frozen by three different techniques and relationship with subsequent fertility in vivo. Cryobiology.

[24] Tselutin K, Seigneurin F, Blesbois E (1999). Comparison of cryoprotectants and methods of cryopreservation of fowl spermatozoa. Poult Sci.

[25] Santiago-Moreno J, Castano C, Toledano-Diaz A, Coloma MA, Lopez-Sebastian A, Prieto MT (2011). Semen cryopreservation for the creation of a Spanish poultry breeds cryobank: Optimization of freezing rate and equilibration time. Poult Sci.

[26] Thananurak P, Chuaychu-Noo N, Thelie A, Phasuk Y, Vongpralub T, Blesbois E (2019). Sucrose increases the quality and fertilizing ability of cryopreserved chicken sperms in contrast to raffinose. Poult Sci.

[27] Chauychu-Noo N, Thananurak P, Boonkum W, Vongpralub T, Chankitisakul V (2021). Effect of organic selenium dietary supplementation on quality and fertility of cryopreserved chicken sperm. Cryobiology.

[28] Murugesan S, Mahapatra R (2020). Cryopreservation of Ghagus chicken semen: Effect of cryoprotectants, diluents and thawing temperature. Reprod Domest Anim.

[29] Long JA, Kulkarni G (2004). An effective method for improving the fertility of glycerol-exposed poultry semen. Poult Sci.

[30] Zong Y, Sun Y, Li Y, Mehaisen GMK, Yuan J, Ma H, et al. Effect of glycerol concentration, glycerol removal method, and straw type on the quality and fertility of frozen chicken semen. Poult Sci. 2022;101(6):101840. 10.1016/j.psj.2022.101840. 10.1016/j.psj.2022.101840 PMC9018144 35413595

[31] Moce E, Grasseau I, Blesbois E (2010). Cryoprotectant and freezing-process alter the ability of chicken sperm to acrosome react. Anim Reprod Sci.

[32] Abouelezz FM, Castano C, Toledano-Diaz A, Esteso MC, Lopez-Sebastian A, Campo JL (2015). Sperm-egg penetration assay assessment of the contraceptive effects of glycerol and egg yolk in rooster sperm diluents. Theriogenology.

[33] Neville WJ, Macpherson JW, Reinhart B (1971). The contraceptive action of glycerol in chickens. Poult Sci.

[34] Purdy PH, Song Y, Silversides FG, Blackburn HD (2009). Evaluation of glycerol removal techniques, cryoprotectants, and insemination methods for cryopreserving rooster sperm with implications of regeneration of breed or line or both. Poult Sci.

[35] Mehdipour M, Daghigh KH, Martinez-Pastor F (2020). Poloxamer 188 exerts a cryoprotective effect on rooster sperm and allows decreasing glycerol concentration in the freezing extender. Poult Sci.

[36] Hammerstedt RH, Graham JK (1992). Cryopreservation of poultry sperm: The enigma of glycerol. Cryobiology.

[37] Woelders H, Zuidberg CA, Hiemstra SJ (2006). Animal genetic resources conservation in the Netherlands and Europe: Poultry perspective. Poult Sci.

[38] Behnamifar A, Bernal B, Torres O, Luis-Chincoya H, GGil M, García-Casado P, et al. Research Note: Evaluation of two methods for adding cryoprotectant to semen and effects of bovine serum albumin on quality characteristics of cryopreserved rooster spermatozoa. Poult Sci. 2021;100(6):101093. 10.1016/j.psj.2021.101093. 10.1016/j.psj.2021.101093 PMC8120946 33965806

[39] Tang M, Cao J, Yu Z, Liu H, Yang F, Huang S, et al. New semen freezing method for chicken and drake using dimethylacetamide as the cryoprotectant. Poult Sci. 2021;100(8):101091. 10.1016/j.psj.2021.101091. 10.1016/j.psj.2021.101091 PMC8260864 34225205

[40] Rakha BA, Ansari MS, Akhter S, Zafar Z, Hussain I, Santiago-Moreno J (2017). Cryopreservation of Indian red jungle fowl (Gallus gallus murghi) semen with polyvinylpyrrolidone. Cryobiology.

[41] Rakha BA, Ansari MS, Akhter S, Santiago-Moreno J, Blesbois E (2018). Cryoprotectant effects of egg yolk on Indian red jungle fowl (Gallus gallus murghi) sperm. Theriogenology.

[42] Mehdipour M, Daghigh KH, Moghaddam G, Hamishehkar H (2018). Effect of egg yolk plasma and soybean lecithin on rooster frozen-thawed sperm quality and fertility. Theriogenology.

[43] Sun L, He M, Wu C, Zhang S, Dai J, Zhang D. Beneficial influence of soybean lecithin nanoparticles on rooster frozen–thawed semen quality and fertility. Animals (Basel). 2021;11:1169. 10.3390/ani11061769 PMC8231592 34199159

[44] Chatterjee S, Gagnon C (2001). Production of reactive oxygen species by spermatozoa undergoing cooling, freezing, and thawing. Mol Reprod Dev.

[45] Partyka A, Lukaszewicz E, Nizanski W (2012). Effect of cryopreservation on sperm parameters, lipid peroxidation and antioxidant enzymes activity in fowl semen. Theriogenology.

[46] Surai PF, Cerolini S, Wishart GJ, Speake BK, Sparks NHC (1998). Lipid and antioxidant composition of chicken semen and its susceptibility to peroxidation. Avian Poult Biol Rev.

[47] Koppers AJ, De Iuliis GN, Finnie JM, McLaughlin EA, Aitken RJ (2008). Significance of mitochondrial reactive oxygen species in the generation of oxidative stress in spermatozoa. J Clin Endocrinol Metab.

[48] Castro LS, Hamilton TR, Mendes CM, Nichi M, Barnabe VH, Visintin JA (2016). Sperm cryodamage occurs after rapid freezing phase: Flow cytometry approach and antioxidant enzymes activity at different stages of cryopreservation. J Anim Sci Biotechnol.

[49] Surai PF, Fujihara N, Speake BK, Brillard J, Wishart GJ, Sparks NHC (2001). Polyunsaturated fatty acids, lipid peroxidation and antioxidant protection in avian semen - Review -. Asian-Australas J Anim Sci.

[50] Partyka A, Nizanski W (2021). Supplementation of avian semen extenders with antioxidants to improve semen Quality-Is it an effective strategy?. Antioxidants (Basel).

[51] Siari S, Mehri M, Sharafi M (2022). Supplementation of Beltsville extender with quercetin improves the quality of frozen-thawed rooster semen. Br Poult Sci.

[52] Masoudi R, Asadzadeh N, Sharafi M. Effects of freezing extender supplementation with mitochondria-targeted antioxidant Mito-TEMPO on frozen-thawed rooster semen quality and reproductive performance. Anim Reprod Sci. 2021;225:106671. 10.1016/j.anireprosci.2020.106671. 10.1016/j.anireprosci.2020.106671 33340960

[53] Thananurak P, Chuaychu-Noo N, Thelie A, Phasuk Y, Vongpralub T, Blesbois E (2020). Different concentrations of cysteamine, ergothioneine, and serine modulate quality and fertilizing ability of cryopreserved chicken sperm. Poult Sci.

[54] Amini MR, Kohram H, Zare-Shahaneh A, Zhandi M, Sharideh H, Nabi MM (2015). The effects of different levels of catalase and superoxide dismutase in modified Beltsville extender on rooster post-thawed sperm quality. Cryobiology.

[55] Masoudi R, Sharafi M, Zare SA, Kohram H, Nejati-Amiri E, Karimi H (2018). Supplementation of extender with coenzyme Q10 improves the function and fertility potential of rooster spermatozoa after cryopreservation. Anim Reprod Sci.

[56] Mehaisen G, Partyka A, Ligocka Z, Nizanski W. Cryoprotective effect of melatonin supplementation on post-thawed rooster sperm quality. Anim Reprod Sci. 2020;212:106238. 10.1016/j.anireprosci.2019.106238. 10.1016/j.anireprosci.2019.106238 31864488

[57] Appiah MO, He B, Lu W, Wang J (2019). Antioxidative effect of melatonin on cryopreserved chicken semen. Cryobiology.

[58] Fattah A, Sharafi M, Masoudi R, Shahverdi A, Esmaeili V, Najafi A (2017). L-Carnitine in rooster semen cryopreservation: Flow cytometric, biochemical and motion findings for frozen-thawed sperm. Cryobiology.

[59] Lotfi S, Mehri M, Sharafi M, Masoudi R (2017). Hyaluronic acid improves frozen-thawed sperm quality and fertility potential in rooster. Anim Reprod Sci.

[60] Moghbeli M, Kohram H, Zare-Shahaneh A, Zhandi M, Sharafi M, Nabi MM (2016). Are the optimum levels of the catalase and vitamin E in rooster semen extender after freezing-thawing influenced by sperm concentration?. Cryobiology.

[61] Najafi D, Taheri RA, Najafi A, Rouhollahi AA, Alvarez-Rodriguez M (2018). Effect of Achillea millefolium-loaded nanophytosome in the post-thawing sperm quality and oxidative status of rooster semen. Cryobiology.

[62] Partyka A, Nizanski W, Bajzert J, Lukaszewicz E, Ochota M (2013). The effect of cysteine and superoxide dismutase on the quality of post-thawed chicken sperm. Cryobiology.

[63] Chuaychu-Noo N, Thananurak P, Chankitisakul V, Vongpralub T (2017). Supplementing rooster sperm with Cholesterol-Loaded-Cyclodextrin improves fertility after cryopreservation. Cryobiology.

[64] Kaka A, Wahid H, Rosnina Y, Yimer N, Khumran AM, Sarsaifi K (2015). Alpha-Linolenic acid supplementation in BioXcell(R) extender can improve the quality of post-cooling and frozen-thawed bovine sperm. Anim Reprod Sci.

[65] Briard JG, Poisson JS, Turner TR, Capicciotti CJ, Acker JP, Ben RN (2016). Small molecule ice recrystallization inhibitors mitigate red blood cell lysis during freezing, transient warming and thawing. Sci Rep.

[66] Wang Z, Yang B, Chen Z, Liu D, Jing L, Gao C (2020). Bioinspired cryoprotectants of Glucose-Based carbon dots. ACS Appl Bio Mater.

[67] Bai G, Song Z, Geng H, Gao D, Liu K, Wu S (2017). Oxidized Quasi-Carbon nitride quantum dots inhibit ice growth. Adv Mater.

[68] Robles V, Valcarce DG, Riesco MF (2019). The use of antifreeze proteins in the cryopreservation of gametes and embryos. Biomolecules.

[69] Mehdipour M, Daghigh-Kia H, Najafi A, Martinez-Pastor F. Type III antifreeze protein (AFP) improves the post-thaw quality and in vivo fertility of rooster spermatozoa. Poult Sci. 2021;100(8):101291. 10.1016/j.psj.2021.101291. 10.1016/j.psj.2021.101291 PMC8260870 34217904

[70] Cheng CY, Chen PR, Chen CJ, Wang SH, Chen CF, Lee YP (2015). Differential protein expression in chicken spermatozoa before and after freezing-thawing treatment. Anim Reprod Sci.

[71] Qi XL, Xing K, Huang Z, Chen Y, Wang L, Zhang LC (2020). Comparative transcriptome analysis digs out genes related to antifreeze between fresh and frozen-thawed rooster sperm. Poult Sci.

[72] Long JA, Bongalhardo DC, Pelaez J, Saxena S, Settar P, O'Sullivan NP (2010). Rooster semen cryopreservation: Effect of pedigree line and male age on postthaw sperm function. Poult Sci.

[73] Santiago-Moreno J, Bernal B, Perez-Cerezales S, Castano C, Toledano-Diaz A, Esteso MC, et al. Seminal plasma amino acid profile in different breeds of chicken: Role of seminal plasma on sperm cryoresistance. PLoS ONE. 2019;14(1):e209910. 10.1371/journal.pone.0209910. 10.1371/journal.pone.0209910 PMC6319765 30608977

[74] El-Sheshtawy RI (2021). Effect of Tris-extender supplemented with a combination of turmeric and ethylene glycol on buffalo bull semen freezability and in vivo fertility. Trop Anim Health Prod.

[75] Yeste M, Estrada E, Casas I, Bonet S, Rodriguez-Gil JE (2013). Good and bad freezability boar ejaculates differ in the integrity of nucleoprotein structure after freeze-thawing but not in ROS levels. Theriogenology.

[76] Sztein JM, Takeo T, Nakagata N (2018). History of cryobiology, with special emphasis in evolution of mouse sperm cryopreservation. Cryobiology.

[77] Mitchell RL, Buckland RB, Kennedy BW (1977). Heritability of fertility of frozen and fresh chicken semen and the relationship between the fertility of frozen and fresh semen. Poult Sci.

[78] Bernal B, Iglesias-Cabeza N, Sanchez-Rivera U, Toledano-Diaz A, Castano C, Perez-Cerezales S (2020). Effect of supplementation of valine to chicken extender on sperm cryoresistance and post-thaw fertilization capacity. Poult Sci.

[79] Ribeiro JC, Carrageta DF, Bernardino RL, Alves MG, Oliveira PF (2022). Aquaporins and animal gamete cryopreservation: Advances and future challenges. Animals (Basel).

[80] Khan IM, Cao Z, Liu H, Khan A, Rahman SU, Khan MZ (2021). Impact of cryopreservation on spermatozoa freeze-thawed traits and relevance OMICS to assess sperm Cryo-Tolerance in farm animals. Front Vet Sci.

[81] Seigneurin F, Grasseau I, Chapuis H, Blesbois E (2013). An efficient method of guinea fowl sperm cryopreservation. Poult Sci.

[82] Iaffaldano N, Romagnoli L, Manchisi A, Rosato MP (2011). Cryopreservation of turkey semen by the pellet method: Effects of variables such as the extender, cryoprotectant concentration, cooling time and warming temperature on sperm quality determined through principal components analysis. Theriogenology.

[83] Blackburn HD, Silversides F, Purdy PH (2009). Inseminating fresh or cryopreserved semen for maximum efficiency: Implications for gene banks and industry 1. Poult Sci.

[84] Blesbois E, Seigneurin F, Grasseau I, Limouzin C, Besnard J, Gourichon D (2007). Semen cryopreservation for ex situ management of genetic diversity in chicken: Creation of the French avian cryobank. Poult Sci.

[85] Blackburn HD (2006). The National Animal Germplasm Program: Challenges and opportunities for poultry genetic resources. Poult Sci.

[86] Silversides FG, Purdy PH, Blackburn HD (2012). Comparative costs of programmes to conserve chicken genetic variation based on maintaining living populations or storing cryopreserved material. Br Poult Sci.

[87] Nakamura Y, Kagami H, Tagami T (2013). Development, differentiation and manipulation of chicken germ cells. Dev Growth Differ.

[88] Paiva SR, Mcmanus CM, Blackburn H (2016). Conservation of animal genetic resources – a new tact. Livest Sci.

[89] Tajima A (2002). Production of germ-line chimeras and their application in domestic chicken. Avian Poult Biol Rev.

[90] Petitte JN, Clark ME, Liu G, Gibbins A, Etches RJ (1990). Production of somatic and germline chimeras in the chicken by transfer of early blastodermal cells. Development.

[91] Tajima A, Naito M, Yasuda Y, Kuwana T (1993). Production of germ line chimera by transfer of primordial germ cells in the domestic chicken (Gallus domesticus). Theriogenology.

[92] Tonus C, Connan D, Waroux O, Vandenhove B, Wayet J, Gillet L (2017). Cryopreservation of chicken primordial germ cells by vitrification and slow freezing: A comparative study. Theriogenology.

[93] Tajima A, Hayashi H, Kamizumi A, Ogura J, Kuwana T, Chikamune T (1999). Study on the concentration of circulating primordial germ cells (cPGCs) in early chick embryos. J Exp Zool.

[94] Mozdziak PE, Angerman-Stewart J, Rushton B, Pardue SL, Petitte JN (2005). Isolation of chicken primordial germ cells using fluorescence-activated cell sorting. Poult Sci.

[95] Han JY, Park TS, Hong YH, Jeong DK, Kim JN, Kim KD (2002). Production of germline chimeras by transfer of chicken gonadal primordial germ cells maintained in vitro for an extended period. Theriogenology.

[96] Tajima A, Naito M, Yasuda Y, Kuwana T (1998). Production of germ-line chimeras by transfer of cryopreserved gonadal primordial germ cells (gPGCs) in chicken. J Exp Zool.

[97] Yuki N, Takeo M, Mitsuru N, Atsushi T (2015). A new method for isolating viable gonadal germ cells from 7-day-old chick embryos. J Poult Sci.

[98] Chaipipat S, Prukudom S, Sritabtim K, Kuwana T, Piyasanti Y, Sinsiri R (2021). Primordial germ cells isolated from individual embryos of red junglefowl and indigenous pheasants of Thailand. Theriogenology.

[99] Hu T, Taylor L, Sherman A, Keambou TC, Kemp SJ, Whitelaw B, et al. A low-tech, cost-effective and efficient method for safeguarding genetic diversity by direct cryopreservation of poultry embryonic reproductive cells. Elife. 2022;11:e74036. 10.7554/eLife.74036. 10.7554/eLife.74036 PMC8789256 35074046

[100] Tsunekawa N, Naito M, Sakai Y, Nishida T, Noce T (2000). Isolation of chicken vasa homolog gene and tracing the origin of primordial germ cells. Development.

[101] Szczerba A, Kuwana T, Bednarczyk M (2021). Concentration and total number of circulating primordial germ cells in Green-legged Partridgelike chicken embryos. Poult Sci.

[102] Macdonald J, Glover JD, Taylor L, Sang HM, McGrew MJ. Characterisation and germline transmission of cultured avian primordial germ cells. PLoS ONE. 2010;5(11):e15518. 10.1371/journal.pone.0015518. 10.1371/journal.pone.0015518 PMC2993963 21124737

[103] Woodcock ME, Gheyas AA, Mason AS, Nandi S, Taylor L, Sherman A (2019). Reviving rare chicken breeds using genetically engineered sterility in surrogate host birds. Proc Natl Acad Sci U S A.

[104] Nandi S, Whyte J, Taylor L, Sherman A, Nair V, Kaiser P (2016). Cryopreservation of specialized chicken lines using cultured primordial germ cells. Poult Sci.

[105] Lin M, Thorne MH, Martin IC, Sheldon BL, Jones RC (1995). Development of the gonads in the triploid (ZZW and ZZZ) fowl, Gallus domesticus, and comparison with normal diploid males (ZZ) and females (ZW). Reprod Fertil Dev.

[106] Naito M, Tajima A, Yasuda Y, Kuwana T (1994). Production of germline chimeric chickens, with high transmission rate of donor-derived gametes, produced by transfer of primordial germ cells. Mol Reprod Dev.

[107] Nakamura Y, Usui F, Ono T, Takeda K, Nirasawa K, Kagami H (2010). Germline replacement by transfer of primordial germ cells into partially sterilized embryos in the chicken. Biol Reprod.

[108] Nakamura Y, Usui F, Miyahara D, Mori T, Ono T, Kagami H (2012). X-irradiation removes endogenous primordial germ cells (PGCs) and increases germline transmission of donor PGCs in chimeric chickens. J Reprod Dev.

[109] Molnar M, Lazar B, Sztan N, Vegi B, Drobnyak A, Toth R (2019). Investigation of the Guinea fowl and domestic fowl hybrids as potential surrogate hosts for avian cryopreservation programmes. Sci Rep.

[110] Ballantyne M, Woodcock M, Doddamani D, Hu T, Taylor L, Hawken RJ (2021). Direct allele introgression into pure chicken breeds using Sire Dam Surrogate (SDS) mating. Nat Commun.

[111] Nakamura Y (2016). Poultry genetic resource conservation using primordial germ cells. J Reprod Dev.

[112] Yu F, Zhu Z, Chen X, Huang J, Jia R, Pan J (2019). Isolation, characterization and germline chimera preparation of primordial germ cells from the Chinese Meiling chicken. Poult Sci.

[113] Lazar B, Molnar M, Sztan N, Vegi B, Drobnyak A, Toth R, et al. Successful cryopreservation and regeneration of a partridge colored Hungarian native chicken breed using primordial germ cells. Poult Sci. 2021;100(8):101207. 10.1016/j.psj.2021.101207. 10.1016/j.psj.2021.101207 PMC8271167 34242944

[114] Ballantyne M, Taylor L, Hu T, Meunier D, Nandi S, Sherman A, et al. Avian primordial germ cells are bipotent for male or female gametogenesis. Front Cell Dev Biol. 2021;9:726827. 10.3389/fcell.2021.726827. 10.3389/fcell.2021.726827 PMC8511492 34660583

[115] Tagami T, Kagami H, Matsubara Y, Harumi T, Naito M, Takeda K (2007). Differentiation of female primordial germ cells in the male testes of chicken (Gallus gallus domesticus). Mol Reprod Dev.

[116] Sekita Y, Nakamura T, Kimura T (2016). Reprogramming of germ cells into pluripotency. World J Stem Cells.

[117] Amini MJ, Sabzalipoor H, Kehtari M, Enderami SE, Soleimani M, Nikzad H (2018). Derivation of male germ cells from induced pluripotent stem cells by inducers: A review. Cytotherapy.

[118] Ryder OA, Onuma M (2018). Viable cell culture banking for biodiversity characterization and conservation. Annu Rev Anim Biosci.

[119] Oikawa M, Kobayashi H, Sanbo M, Mizuno N, Iwatsuki K, Takashima T (2022). Functional primordial germ cell-like cells from pluripotent stem cells in rats. Science.

[120] Guan J, Wang G, Wang J, Zhang Z, Fu Y, Cheng L (2022). Chemical reprogramming of human somatic cells to pluripotent stem cells. Nature.

[121] Zhao R, Zuo Q, Yuan X, Jin K, Jin J, Ding Y (2021). Production of viable chicken by allogeneic transplantation of primordial germ cells induced from somatic cells. Nat Commun.

[122] FAO. Cryoconservation of animal genetic resources. FAO Animal Production and Health Guidelines No. 12. Rome; 2012.

[123] Santiago-Moreno J, Blesbois E. Animal board invited review: Germplasm technologies for use with poultry. Animal. 2022;16(3):100475. 10.1016/j.animal.2022.100475. 10.1016/j.animal.2022.100475 35220173

[124] Song Y, Silversides F (2007). Heterotopic transplantation of testes in newly hatched chickens and subsequent production of offspring via intramagnal insemination. Biol Reprod.

[125] Song Y, Silversides FG (2007). Production of offspring from cryopreserved chicken testicular tissue. Poult Sci.

[126] Silversides FG, Robertson MC, Liu J (2013). Growth of subcutaneous chicken testicular transplants. Poult Sci.

[127] Liu J, Cheng KM, Silversides FG (2013). Production of live offspring from testicular tissue cryopreserved by vitrification procedures in Japanese quail (Coturnix japonica). Biol Reprod.

[128] Benesova B, Mucksova J, Kalina J, Trefil P (2014). Restoration of spermatogenesis in infertile male chickens after transplantation of cryopreserved testicular cells. Br Poult Sci.

[129] Song Y, Silversides FG (2006). The technique of orthotopic ovarian transplantation in the chicken. Poult Sci.

[130] Kosenko OV (2007). Orthotopic transplantation of donor ovary as an alternative method of artificial reproduction of fowl. Russ Agricult Sci.

[131] Silversides FG, Song Y, Renema R, Rathgeber BR, Classen HL (2008). Cryopreservation of germplasm from chickens kept in Canadian research institutions. Can J Anim Sci.

[132] Silversides FG, Robertson MC, Liu J (2013). Cryoconservation of avian gonads in Canada. Poult Sci.

[133] Liu J, Elsasser TH, Long JA (2017). Microscopic morphology and apoptosis of ovarian tissue after cryopreservation using a vitrification method in post-hatching turkey poults. Meleagris gallopavo J Poult Sci.

[134] Liu J, Cheng KM, Silversides FG (2012). Novel needle-in-straw vitrification can effectively preserve the follicle morphology, viability, and vascularization of ovarian tissue in Japanese quail (Coturnix japonica). Anim Reprod Sci.

[135] Liu J, Song Y, Cheng KM, Silversides FG (2010). Production of donor-derived offspring from cryopreserved ovarian tissue in Japanese quail (Coturnix japonica). Biol Reprod.

[136] Song Y, Cheng KM, Robertson MC, Silversides FG (2012). Production of donor-derived offspring after ovarian transplantation between Muscovy (Cairina moschata) and Pekin (Anas platyrhynchos) ducks. Poult Sci.

[137] Liptoi K, Buda K, Rohn E, Drobnyak A, Meleg EE, Palinkas-Bodzsar N, et al. Improvement of the application of gonadal tissue allotransplantation in the in vitro conservation of chicken genetic lines. Anim Reprod Sci. 2020;213:106280. 10.1016/j.anireprosci.2020.106280. 10.1016/j.anireprosci.2020.106280 31987330

[138] Song Y, Silversides FG (2007). Offspring produced from orthotopic transplantation of chicken ovaries. Poult Sci.

[139] Liptoi K, Horvath G, Gal J, Varadi E, Barna J (2013). Preliminary results of the application of gonadal tissue transfer in various chicken breeds in the poultry gene conservation. Anim Reprod Sci.

[140] Song Y, Silversides FG (2008). Long-term production of donor-derived offspring from chicken ovarian transplants. Poult Sci.

[141] Fogarty NM, Maxwell WM, Eppleston J, Evans G (2000). The viability of transferred sheep embryos after long-term cryopreservation. Reprod Fertil Dev.

[142] Carwell DB, Pitchford JA, Gentry GT, Blackburn H, Bondioli KR, Godke RA (2009). Beef cattle pregnancy rates following insemination with aged frozen Angus semen. Reprod Fert Develop.

[143] Hezavehei M, Sharafi M, Kouchesfahani HM, Henkel R, Agarwal A, Esmaeili V (2018). Sperm cryopreservation: A review on current molecular cryobiology and advanced approaches. Reprod Biomed Online.

[144] Perez-Cerezales S, Gutierrez-Adan A, Martinez-Paramo S, Beirao J, Herraez MP (2011). Altered gene transcription and telomere length in trout embryo and larvae obtained with DNA cryodamaged sperm. Theriogenology.

[145] Eastick J, Venetis C, Cooke S, Storr A, Susetio D, Chapman M (2017). Is early embryo development as observed by time-lapse microscopy dependent on whether fresh or frozen sperm was used for ICSI? A cohort study. J Assist Reprod Genet.

[146] Salehi M, Mahdavi AH, Sharafi M, Shahverdi A (2020). Cryopreservation of rooster semen: Evidence for the epigenetic modifications of thawed sperm. Theriogenology.

[147] Kopeika J, Thornhill A, Khalaf Y (2015). The effect of cryopreservation on the genome of gametes and embryos: Principles of cryobiology and critical appraisal of the evidence. Hum Reprod Update.

[148] Blackburn HD (2018). Biobanking genetic material for agricultural animal species. Annu Rev Anim Biosci.

[149] Blackburn HD (2004). Development of national animal genetic resource programs. Reprod Fertil Dev.

[150] Shepherd RK, Woolliams JA (2004). Minimising inbreeding in small populations by rotational mating with frozen semen. Genet Res.

[151] Sonesson AK, Goddard ME, Meuwissen TH (2002). The use of frozen semen to minimize inbreeding in small populations. Genet Res.

[152] Grégoire L, Coralie D, Etienne V (2011). Impact of the use of cryobank samples in a selected cattle breed: A simulation study. Gene Sel Evol.

[153] Holt WV, Comizzoli P. Opportunities and limitations for reproductive science in species conservation. Annu Rev Anim Biosci. 2021;11:55 10.1146/annurev-animal-013120-030858 34699258

[154] Leroy G, Boettcher P, Besbes B, Danchin-Burge C, Hiemstra SJ (2019). Cryoconservation of animal genetic resources in Europe and two African countries: A gap analysis. Diversity.

